# Antibody Persistence 6 Months Post-Vaccination with BNT162b2 among Health Care Workers

**DOI:** 10.3390/vaccines9101125

**Published:** 2021-10-03

**Authors:** Flaminia Campo, Aldo Venuti, Fulvia Pimpinelli, Elva Abril, Giovanni Blandino, Laura Conti, Armando De Virgilio, Federico De Marco, Vincenzo Di Noia, Enea Gino Di Domenico, Simona Di Martino, Fabrizio Ensoli, Diana Giannarelli, Chiara Mandoj, Francesco Mazzola, Silvia Moretto, Gerardo Petruzzi, Fabrizio Petrone, Barbara Pichi, Martina Pontone, Antonello Vidiri, Branka Vujovic, Giulia Piaggio, Eleonora Sperandio, Valentina Rosati, Francesco Cognetti, Aldo Morrone, Gennaro Ciliberto, Raul Pellini

**Affiliations:** 1 Department Otolaryngology Head and Neck Surgery, IRCCS Regina Elena National Cancer Institute, Istituti Fisioterapici Ospitalieri (IFO), 00144 Rome, Italy; flaminiacampo@gmail.com (F.C.); francesco.mazzola@ifo.gov.it (F.M.); silvia.moretto@ifo.gov.it (S.M.); gerardo.petruzzi@ifo.gov.it (G.P.); barbara.pichi@ifo.gov.it (B.P.); valentina_rosati@hotmail.it (V.R.); Raul.pellini@ifo.gov.it (R.P.); 2 HPV Unit, UOSD Tumor Immunology and Immunotherapy, IRCCS Regina Elena National Cancer Institute, Istituti Fisioterapici Ospitalieri (IFO), 00144 Rome, Italy; 3 Department of Microbiology and Virology, IRCCS San Gallicano Dermatological Institute, Istituti Fisioterapici Ospitalieri (IFO), 00144 Rome, Italy; fulvia.pimpinelli@ifo.gov.it (F.P.); elva.abril@ifo.gov.it (E.A.); enea.didomenico@ifo.gov.it (E.G.D.D.); fabrizio.ensoli@ifo.gov.it (F.E.); martina.pontone@ifo.gov.it (M.P.); 4 Oncogenomic and Epigenetic Unit, IRCCS Regina Elena National Cancer Institute, Istituti Fisioterapici Ospitalieri (IFO), 00144 Rome, Italy; giovanni.blandino@ifo.gov.it; 5 Department Clinical Pathology and Cancer Biobank, IRCCS Regina Elena National Cancer Institute, Istituti Fisioterapici Ospitalieri (IFO), 00144 Rome, Italy; laura.conti@ifo.gov.it (L.C.); Chiara.mandoj@ifo.gov.it (C.M.); 6 Department of Biomedical Sciences, Humanitas University, Via Rita Levi Montalcini 4, Pieve Emanuele, 20072 Milan, Italy; armando.devirgilio@gmail.com; 7 IRCCS Humanitas Research Hospital, Via Manzoni 56, Rozzano, 20089 Milan, Italy; 8 RIDAIT Department, IRCCS Regina Elena National Cancer Institute, Istituti Fisioterapici Ospitalieri (IFO), 00144 Rome, Italy; federico.demarco@ifo.gov.it; 9 Medical Oncology, IRCCS Regina Elena National Cancer Institute, 00144 Rome, Italy; vincenzo.dinoia@ifo.gov.it (V.D.N.); francesco.cognetti@ifo.gov.it (F.C.); 10 Department of Pathology, IRCCS Regina Elena National Cancer Institute, Istituti Fisioterapici Ospitalieri (IFO), 00144 Rome, Italy; simona.dimartino@ifo.gov.it; 11 Biostatistical Unit, IRCCS Regina Elena National Cancer Institute, Istituti Fisioterapici Ospitalieri (IFO), 00144 Rome, Italy; diana.giannarelli@ifo.gov.it; 12 U.O.C. DITRAR, IRCCS Regina Elena National Cancer Institute, Istituti Fisioterapici Ospitalieri (IFO), 00144 Rome, Italy; fabrizio.petrone@ifo.gov.it; 13 Department of Radiology and Diagnostic Imaging, IRCCS Regina Elena National Cancer Institute, Istituti Fisioterapici Ospitalieri (IFO), 00144 Rome, Italy; antonello.vidiri@ifo.gov.it; 14 Medical Direction, IRCCS Regina Elena National Cancer Institute and San Gallicano Institute, 00144 Rome, Italy; branka.vujovic@ifo.gov.it; 15 Department of Research, IRCSS Technological Innovation & Advanced Diagnostics, Regina Elena National Cancer Institute, 00144 Rome, Italy; giulia.piaggio@ifo.gov.it; 16 UOSD Clinical Trial Center, Biostatistic and Bionformatic, IRCCS Regina Elena National Cancer Institute, Istituti Fisioterapici Ospitalieri (IFO), 00144 Rome, Italy; eleonora.sperandio@ifo.gov.it; 17 Scientific Direction, IRCCS San Gallicano Dermatological Institute, Istituti Fisioterapici Ospitalieri (IFO), 00144 Rome, Italy; Aldo.morrone@ifo.gov.it; 18 Scientific Direction, IRCCS Regina Elena National Cancer Institute, Istituti Fisioterapici Ospitalieri (IFO), 00144 Rome, Italy; gennaro.ciliberto@ifo.gov.it

**Keywords:** COVID-19, SARS-CoV-2, mRNA vaccine, BNT162b2, antibody response

## Abstract

Background: We present immunogenicity data 6 months after the first dose of BNT162b2 in correlation with age, gender, BMI, comorbidities and previous SARS-CoV-2 infection. Methods: An immunogenicity evaluation was carried out among health care workers (HCW) vaccinated at the Istituti Fisioterapici Ospitalieri (IFO). All HCW were asked to be vaccine by the national vaccine campaign at the beginning of 2021. Serum samples were collected on day 1 just prior to the first dose of the vaccine and on day 21 just prior to the second vaccination dose. Thereafter sera samples were collected 28, 49, 84 and 168 days after the first dose of BNT162b2. Quantitative measurement of IgG antibodies against S1/S2 antigens of SARS-CoV-2 was performed with a commercial chemiluminescent immunoassay. Results: Two hundred seventy-four HWCs were analyzed, 175 women (63.9%) and 99 men (36.1%). The maximum antibody geometric mean concentration (AbGMC) was reached at T2 (299.89 AU/mL; 95% CI: 263.53–339.52) with a significant increase compared to baseline (*p* < 0.0001). Thereafter, a progressive decrease was observed. At T5, a median decrease of 59.6% in COVID-19 negative, and of 67.8% in COVID-19 positive individuals were identified with respect to the highest antibody response. At T1, age and previous COVID-19 were associated with differences in antibody response, while at T2 and T3 differences in immune response were associated with age, gender and previous COVID-19. At T4 and T5, only COVID-19 positive participants demonstrated a greater antibody response, whereas no other variables seemed to influence antibody levels. Conclusions: Overall our study clearly shows antibody persistence at 6 months, albeit with a certain decline. Thus, the use of this vaccine in addressing the COVID-19 pandemic is supported by our results that in turn open debate about the need for further boosts.

## 1. Introduction

Among vaccines against severe acute respiratory syndrome coronavirus 2 (SARS-CoV-2), messenger RNA (mRNA) vaccines represent great promise for the decrease in the spread of infection. However, the U.S. Food and Drug Administration and the European Medicines Agency approved the SARS-CoV-2 vaccination program in an emergency, and presently there is a lack of data on the length of the immune response. An optimal COVID-19 vaccine should provide a long-lasting antibody response and would stimulate sterilizing immunity to avoid disease and forward transmission [1]. Notwithstanding rigorous research, it is not usually predictable the kinetics, and evolution of immune memory to infection or vaccine based on the initial effector phase [2].

Therefore, a longer follow-up is needed to assess the antibody kinetics in individuals after a two-dose regimen of BNT162b2. These data are important to program future vaccination campaigns. We present the experience with BNT162b2 vaccination in 274 participants, an ongoing longitudinal observational study of health-care workers (HCWs) in Istituti Fisioterapici Ospitalieri of Rome [3,4]. Here, we update immunogenicity data 6 months after the first vaccine dose in correlation with age, gender, BMI, comorbidities and previous SARS-CoV-2 infection.

## 2. Materials and Methods

### 2.1. Study Design and Participants

A collaborative team carried out an immunogenicity evaluation among HCWs having received two doses of the BNT162b2 vaccine at the Istituti Fisioterapici Ospitalieri (IFO). Briefly, the study protocol complied with the tenets of the Helsinki declaration and was approved by the institutional scientific ethics committee (protocol RS1463/21) and registered to a Clinical Trial registry ISRCTN55371988. Participants were requested to provide written informed consent. All the enrolled participants met the following inclusion criteria: (1) provided written informed consent; (2) age between 18–75 years; (3) health workers employed at the Istituti Fisioterapici Ospitalieri (IFO); (4) vaccinated at the Istituti Fisioterapici Ospitalieri (IFO). Key exclusion criteria included: (1) treatment with immunosuppressive therapy; (2) immunosuppression-associated pathology; and (3) pregnancy.

The mRNA vaccine was administered as a 30 microgram/0.3 mL intramuscular injection into the deltoid muscle on days 1 and 21 of the study. A questionnaire to collect socio-demographic and health data was administered to participants at baseline. Serum samples were collected on day 1 just prior to the first dose of vaccine and on day 21 just prior to the second dose vaccination. Thereafter sera samples were collected 28, 49, 84 and 168 days after the first dose of BNT162b2. Collected sera were stored at +4 °C until use and analyzed within 6 h after collection. Aliquots of sera were stored frozen at −20 °C for further analyses. Quantitative measurement of IgG antibodies against S1/S2 antigens of SARS-CoV-2 was performed with a commercial chemiluminescent immunoassay (The LIAISON^®^ SARS-CoV-2 S1/S2 IgG test, Diasorin, Italy) according to the manufacturer’s instruction. The concentration of <3.8 AU/mL was adopted as a cut-off to define negative humoral responses, as reported in the manufacturer instruction.

### 2.2. Statistical Analysis

The Geometric Mean of AU/mL and its 95% confidence interval was described for the total series and for each subgroup. A generalized linear model using the logarithm of titer as a dependent variable was implemented to assess the correlation between gender, age and BMI, comorbidities and seropositive patients with serum concentration. Age was then categorized through quartiles and BMI subgroups were created according to WHO classes as follows: underweight (BMI > 18.5 kg/m^2^), normal weight (BMI ≥ 18.5 = 24.9 kg/m^2^), overweight (BMI ≥ 25 = 29.9 kg/m^2^) and obesity (BMI ≥ 30 kg/m^2^) [5]. However, age and BMI were considered as continuous variables in statistical analysis. The half-life was obtained from the one-compartment modeling which permitted the calculation of the elimination rate of the antibody response.

Statistical analysis was carried out using SPSS Statistics software version 21. A *p* < 0.05 was considered statistically significant.

## 3. Results

Two hundred seventy-four HWCs were analyzed, 175 women (63.9%) and 99 men (36.1%) and all participants were of Caucasian ethnicity. The demographic and clinical characteristics of the HCW population are represented in Table 1. Of these, 15 (5.4%) were categorized as SARS-CoV-2 positive because they reported positive nasopharyngeal swabs for SARS-CoV-2 in previous months. Infections took place between March and November 2020. Too few patients (only 4) reported diabetes and therefore this variable was not further analyzed. Age and BMI were considered as continuous variables in statistical analysis.

All participants still had measurable anti-SARS-CoV-2 S antibodies until day 168.

**Figure 1:**
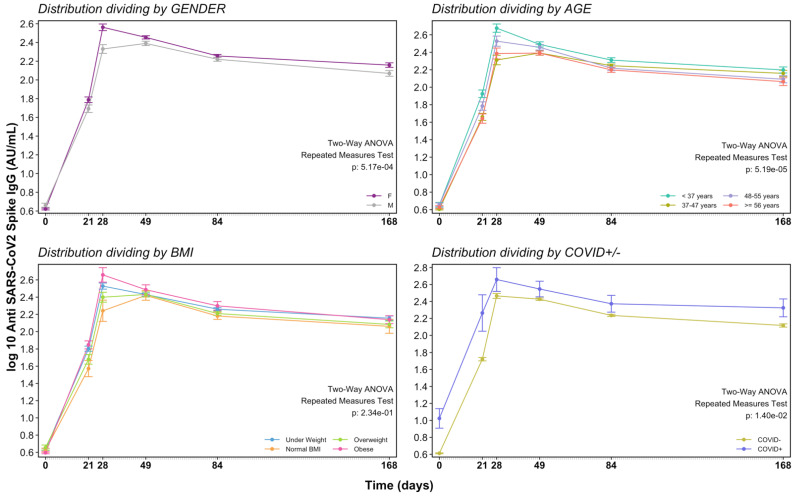
Time course of antibody response after vaccination by gender, age, BMI and previous COVID-19.

At T1, when just one vaccine dose was injected, we already detected a humoral response with AbGMC of 56.69 AU/mL, above the cut-off value. The maximum AbGMC was reached at T2 (299.89 AU/mL; 95% CI: 263.53–339.52) with a significant increase compared to baseline (*p* < 0.0001). Thereafter, a progressive decrease was observed at T3 (271.09 AU/mL; 95% CI: 254.71–289.26), T4 (175.37 AU/mL; 95% CI: 165.51–186.06), and T5 (134.64 AU/mL; 95% CI: 123.25–146.54) (Table 2). At T5, a median decrease of 59.6% in COVID-19 negative, and of 67.8% in COVID-19 positive individuals were identified with respect to the highest antibody response (Figure 1). The estimated half-life of antibodies from data collected until 168 days post-vaccination was 275 days as calculated by the one-compartmental model.

Univariate analysis (Table 3) considering age and BMI as continuous variables showed that at T1, antibody titer was greater in younger, lean, with no hypertension and with having had COVID-19 previously. At T2, a statistically significant difference in antibody levels was observed in the young, lean, female and no hypertension group. At T3, a difference in humoral response was observed only in the younger, in females and in those who had had COVID-19 previously. At T4, antibody titer was greater in the younger people and with those who had previously had COVID-19. Finally, at T5, only people who had previously had COVID-19 were associated with greater humoral response.

Multivariate analysis accounting for potential confounding elements was performed by the inclusion of covariates. Data on multivariate linear regression of AU/mL are reported in Table 3. This analysis demonstrated that at T1 age (*p* = 0.001) and previous COVID-19 (*p* < 0.0001) are statistically associated with differences in antibody response. At T2 and T3, the difference in immune response is associated with age (*p* = 0.0001, *p* = 0.003, respectively), gender (*p* = 0.002, *p* = 0.046, respectively) and previous COVID-19 (*p* = 0.043, *p* = 0.002, respectively). At T4 and T5 only previous COVID-19 is associated with a difference in antibody response (*p* = 0.0001, *p* = 0.002, respectively).

## 4. Discussion

Since the first cases of COVID-19 were described in December 2019 a health emergency with major social and economic disruptions has spread worldwide [6]. Thus, the research of the world scientific community was focused on the development of an effective vaccine.

Notwithstanding rigorous study in humans, kinetic, duration, and evolution of antibody response to immunization are not predictable based on the early effector phase. Therefore, measuring responses over a period of months is essential to determine the durability of the immune response [2,7]. Several recent reports indicate that after two doses of BNT162b2 antibodies persisted up to 3 months, however, a significant decrease in their serum levels could be observed during this period [8,9]. Currently, a longer follow-up after an mRNA vaccine is reported by Doria-Rose et al., showed persistence of antibodies 6 months after the second dose of the mRNA-1273 vaccine in 33 participants included in the phase 1 follow-up of the Moderna study without knowing their initial serological status before vaccination [10]. Our data represent an independent study on antibody levels against S1/S2 SARS-CoV-2 in HCWs 6 months after the first dose of BNT162b2. Notably at T5, 100% of participants demonstrated antigen-specific humoral response with respect to baseline level. At 6 months, a median antibody decreases of 59.6% and 67.8% in COVID-19 negative and COVID-19 positive individuals were identified from the highest antibody response. Favresse et al. reported similar results for COVID-19 negative vs. COVID-19 positive subjects, strengthening our data [9]. Interestingly we reported the correlation of antibody response with multiple variables, however, at present, we do not have a clear hypothesis to explain why different variables were affecting serum level according to time after vaccination. The analysis was performed with age and BMI calculated as continuous variables. Indeed, by aggregating these variables into groups, there is the possibility that valuable information is missing, and analysis findings could be misleading. Furthermore, multivariate linear regression of AU/mL, accounting for potential confounding data by the inclusion of covariates is more insightful and accurate than a univariate analysis. Our data demonstrated statically significant differences by age in antibody response at T1, T2 and T3. It is well recognized in the literature that aging and related immunosenescence may lead to a reduced humoral response to the vaccine [11]. In particular, qualitative differences were observed in the memory B cells and plasma compartment in older adults. This included class switch recombination and differentiation into plasma cells [12,13]. With immunosenescence, another modification is the increase in an inflammatory subset of B cells similar to age-associated B cells (ABCs) as described in mice [14,15]. It was shown that an increased level of CD27 + ABCs in the blood of the elderly was associated with a reduced titer of influenza-specific antibodies [16].

In addition, our observations also showed this difference in vaccine response at T2 and T3, in line with a recent meta-analysis [17] Literature reports advise that COVID-19 exhibits differences in morbidity and mortality between gender. Male patients have almost three times the odds of requiring intensive treatment unit admission and higher odds of death compared to females [18]. Women produce higher antibody titers in response to the trivalent inactivated seasonal influenza vaccination (TIV) [19,20]. More specifically, females achieve equivalent protective antibody titers to males at half the dose of TIV [20], with serum testosterone levels inversely correlating with TIV antibody titers [21]. Finally, several studies have already shown a correlation between the antibody response to the vaccine and the previous infection with SARS-CoV-2.

In the present article, we report that humoral response to the vaccine is statistically correlated with the previous infection in all observed times.

Although the role of Nabs to SARS-CoV-2 is under investigation, measurement of serum neutralizing activity was demonstrated to correlate with protection for other respiratory viruses, such as influenza [22] or respiratory syncytial virus [23]. In this study, we utilized a chemiluminescent immunoassay that detects S1/S2 specific antibodies, but it was not specifically designed to detect Nabs. However, the manufacturer indicates that with 80-AU/mL levels, the probabilities of having plaque reduction neutralization titers of 1:80 and 1:160 were 92% and 87%, respectively [24]. At T5, albeit declining, antibody levels were above >80 AU/mL in a consistent number of enrolled patients (80.7%, 80.5% and 85.8% of the total, COVID-19 negative, and COVID-19 positive participants, respectively), suggesting that a possible neutralizing activity is present 168 days after first vaccine dose.

The principal limitations of the study are the following: (i) our study is a single-center study with a limited number of participants; (ii) all subjects were of Caucasian ethnicity; therefore, it cannot be assumed representative of the general population nor of the non-Caucasian population; (iii). We utilized a questionnaire to collect data on the participants’ socio-demographic and health characteristics, and the possibility of self-reporting bias should be considered; (iv) previous COVID-19 negative HCW did not take into account the possibility of asymptomatic cases; (v) finally, data about cellular immune response and neutralization antibodies are also lacking.

However, a subset of sera from our cohort (175 samples) was utilized as control group in a study on cancer patients and analysed for Nabs. Titre of Nabs and anti-S IgG more than 80 AU/mL were significantly associated (Spearman’s rho = 0.799, *p* < 0.0001). This suggesting that Nabs could be present in sera with a total antibody titer above 80 AU/m [25]. In addition, during follow-up, none of the vaccinee HCW referred any symptoms related to possible COVID-19 disease or positivity to nasopharyngeal swab test.

## 5. Conclusions

Overall, our study clearly shows antibody persistence at 6 months, albeit with a certain decline. Thus, the use of this vaccine in addressing the COVID-19 pandemic is supported by our results that in turn open debate about the need for further boosts.

## Figures and Tables

**Table 1:** Demographic and clinical characteristics of the HCW.

Characteristic						
Sampling	T0	T1	T2	T3	T4	T5
Total Patients	274	271	270	251	250	232
Age						
Median (rage)	46.1 (23–69)	46.1 (23–69)	46.1 (23–69)	45.8 (23–69)	45.8 (23–69)	45.4 (23–69)
Gender						
Female	174	173	170	169	164	156
Male	100	98	100	82	86	76
Bmi						
Under-Weight	20	20	20	19	19	18
Normal-Weight	162	161	160	150	154	141
Pre-Obesity	66	64	64	58	54	52
Obesity	26	26	26	24	23	21
Hypertension						
NO	243	240	239	222	223	202
YES	31	31	31	29	27	30
Hypothyroidism						
NO	256	253	252	233	232	214
YES	18	18	18	18	18	18
Diabetes						
NO	270	267	266	249	248	230
YES	4	4	4	2	2	2
Previous COVID-19						
NO	259	256	255	239	236	218
YES	15	15	15	12	14	14

**Table 2:** GMC and 95% CI for all subjects, COVID-19 negative and COVID-19 positive at T0, T1, T2, T3 T4 and T5 (6 months after first dose).

Characteristic	N. HCW T0 (Day 0)	GMC (95% CI)	N. HCW T1 (Day 21)	GMC (95% CI)	N. HCW T2 (Day 28)	GMC (95% CI)	N. HCW T3 (Day 49)	GMC (95% CI)	N. HCW T4 (Day 84)	GMC (95% CI)	N. HCW T5 (Day 168)	GMC (95% CI)
Sampling		T0		T1		T2		T3		T4		T5
All subjects	274	4.32 (4.14–4.54)	271	56.69 (50.90–63.15	270	299.89 (263.53–339.52)	251	271.09 (254.71–289.26)	250	175.37 (165.51–186.06)	232	134.64 (123.25–146.54)
COVID-19 no	259	4.11 (4.02–4.22)	257	52.51 (47.90–57.67)	256	290.77 (254.38–329.52)	240	266.25 (250.33–283.53)	237	171.47 (162.38–181.34)	219	130.81 (120.45–143.14)
COVID-19 yes	15	10.29 (5.93–17.87)	15	209.13 (83.15–566.19)	15	506.88 (271.74–957.86)	12	388.13 (263.10–600.62)	14	256.17 (166.94–422.14)	14	211.14 (134.33–335.90)

GMC: geometric mean concentration; CI: confidence interval.

**Table 3:** Univariate and multivariate linear regression of antibody levels (AU/mL) by age, BMI, gender, hypertension, hypothyroidism, and previous COVID-19.

		Univariate		Multivariate	
		Beta (95% CI)	*p* Value	Beta (95% CI)	*p* Value
T0	Age (in years)	−0.004 (−0.008; 0.001)	0.054	−0.003 (−0.007; 0.001)	0.092
	Bmi (kg/cm^2^)	0.005 (−0.005; 0.016)	0.299	0.003 (−0.006; 0.013)	0.495
	Gender (male vs. female)	0.093 (0.000; 0.186)	0.050	0.069 (−0.014; 0.149)	0.104
	Hypertension (yes vs. no)	−0.042 (−0.180; 0.096)	0.552	0.037 (−0.086; 0.160)	0.555
	Hypothyroidism (yes vs. no)	−0.018 (−0.199; 0.164)	0.850	0.068 (−0.083; 0.219)	0.379
	Previous COVID-19 (yes vs. no)	0.917 (0.752; 1.083)	0.0001	0.968 (0.799; 1.137)	0.0001
T1	Age (in years)	−0.023 (−0.032; −0.014)	0.0001	−0.016 (−0.026; −0.007)	0.001
	Bmi (kg/cm^2^)	−0.037 (−0.062; −0.013)	0.003	−0.023 (−0.046; 0.001)	0.062
	Gender (male vs. female)	−0.220 (−0.444; 0.005)	0.055	−0.120 (−0.329; 0.090)	0.263
	Hypertension (no vs. yes)	−0.454 (−0.781; −0.127)	0.006	−0.065 (−0.379; 0.249)	0.685
	Hypothyroidism (yes vs. no)	0.116 (−0.319; 0.551)	0.603	0.157 (−0.229; 0.542)	0.426
	Previous COVID-19 (yes vs. no)	1.382 (0.937; 1.826)	0.0001	1.551 (1.120; 1.983)	0.0001
T2	Age (in years)	−0.030 (−0.040; −0.019)	0.0001	−0.023 (−0.035; −0.011)	0.0001
	Bmi (kg/cm^2^)	−0.051 (−0.080; −0.022)	0.001	−0.017 (−0.048; 0.013)	0.257
	Gender (male vs. female)	−0.534 (−0.795; −0.272)	0.0001	−0.422 (−0.686; −0.157)	0.002
	Hypertension (yes vs. no)	−0.605 (−1.005; −0.205)	0.003	−0.123 (−0.533; 0.286)	0.554
	Hypothyroidism (yes vs. no)	0.163 (−0.356; 0.683)	0.538	0.063 (−0.425; 0.552)	0.799
	Previous COVID-19 (yes vs. no)	0.556 (−0.007; 1.118)	0.053	0.565 (0.018; 1.112)	0.043
T3	Age (in years)	−0.009 (−0.014; −0.003)	0.002	−0.009 (−0.015; −0.003)	0.003
	Bmi (kg/cm^2^)	−0.004 (−0.019; 0.011)	0.610	0.006 (−0.010; 0.021)	0.480
	Gender (male vs. female)	−0.148 (−0.282; −0.014)	0.030	−0.140 (−0.278; −0.003)	0.046
	Hypertension (yes vs. no)	−0.065 (−0.263; 0.133)	0.521	0.087 (−0.118; 0.293)	0.404
	Hypothyroidism (yes vs. no)	0.208 (−0.036; 0.452)	0.095	0.216 (−0.022; 0.454)	0.075
	Previous COVID-19 (yes vs. no)	0.377 (0.084; 0.670)	0.012	0.470 (0.171; 0.768)	0.002
T4	Age (in years)	−0.006 (−0.010; −0.001)	0.015	−0.005 (−0.010; 0.001)	0.101
	Bmi (kg/cm^2^)	−0.013 (−0.026; 0.001)	0.064	−0.009 (−0.024; 0.005)	0.206
	Gender (male vs. female)	−0.082 (−0.204; 0.039)	0.186	−0.050 (−0.176; 0.076)	0.435
	Hypertension (yes vs. no)	−0.083 (−0.270; 0.103)	0.381	0.042 (−0.153; 0.237)	0.674
	Hypothyroidism (yes vs. no)	0.036 (−0.188; 0.260)	0.754	0.048 (−0.172; 0.267)	0.670
	Previous COVID-19 (yes vs. no)	0.401 (0.154; 0.649)	0.001	0.474 (0.221; 0.728)	0.0001
T5	Age (in years)	−0.007 (−0.014; 0.001)	0.084	−0.004 (−0.013; 0.004)	0.309
	Bmi (kg/cm^2^)	−0.016 (−0.036; 0.004)	0.116	−0.010 (−0.032; 0.011)	0.355
	Gender (male vs. female)	−0.204 (−0.388; −0.020)	0.031	−0.185 (−0.378; 0.008)	0.061
	Hypertension (yes vs. no)	−0.118 (−0.374; 0.138)	0.367	0.061 (−0.218; 0.340)	0.668
	Hypothyroidism (yes vs. no)	0.189 (−0.136; 0.515)	0.255	0.177 (−0.145; 0.499)	0.281
	Previous COVID-19 (yes vs. no)	0.479 (0.117; 0.840)	0.009	0.605 (0.230; 0.980)	0.002

## Data Availability

Data are going to be added to the clinical trial registry (ISRCTN55371988) where they will be available.
